# The influence of high-fat diet and energy-restricted diet on hematopoietic stem cells: mechanisms and implications

**DOI:** 10.3389/fimmu.2025.1576118

**Published:** 2025-05-30

**Authors:** Zhi’an Chen, Haoyuan Ang, Jie Li, Meiling Yu, Xiaoling Chen, Li Wang

**Affiliations:** Department of Immunology, College of Basic Medicine, Army Medical University (Third Military Medical University), Chongqing, China

**Keywords:** hematopoietic stem cell, high-fat diet, dietary restriction, protein-energy malnutrition, energy

## Abstract

Increasing evidence demonstrates a close relationship between daily diet and homeostasis of the body’s internal environment, particularly hematopoietic system homeostasis. Hematopoietic stem cells (HSCs) are located at the top of the hematopoietic system and have the ability to self-renew and differentiate into various types of immune cells. They play an important role in maintaining body stability and health. Studies have shown that different diets can lead to changes in HSC homeostasis, thereby affecting immune function and overall health status of the body. However, there is a scarcity of comprehensive reviews on how different diets affects HSC function. Therefore, this review summarizes the current progression in research on the effects of a high-fat diet (HFD) and energy-restricted diet on HSC function. HFD has a predominantly negative effect on HSCs, as does severe energy-restricted diet (SERD). Conversely, moderate energy-restricted diet (dietary restriction, DR) promotes the repopulation of HSCs but seriously impairs the differentiation of HSCs into lymphoid lineage. Further study of the influence of different diets on HSCs may aid in designing rational dietary guidelines to optimize the hematopoietic and immune functions of the body, which has significant implications for clinical medical practices.

## 1 Introduction

Hematopoietic stem cells (HSCs), renowned for self-renewal and multilineage differentiation capabilities, are pivotal in sustaining the body’s homeostatic balance. More than 90% of HSCs in adults are in a state of quiescence (1), and most of the hematopoiesis in the body is completed by hematopoietic cells with a higher degree of differentiation. HSCs (Lin^-^Sca1^+^c-Kit^+^) (2) include long-term HSCs (LT-HSCs) and short-term HSCs (ST-HSCs), each with different self-renewal abilities and differentiation potentials. LT-HSCs represent the most primitive state of HSCs, mainly in the cell-cycle-quiescent (G0 phase), and are located at the top of the hematopoietic system with an enduring self-renewal capacity (3). They are essential for maintaining the lifelong activity of the stem HSC pool. LT-HSCs differentiate into ST-HSCs and multipotent progenitors (MPPs). ST-HSCs possess some self-renewal abilities, reconstructing the blood pool and maintaining hematopoiesis, while MPPs cannot rebuild the bone marrow (BM) but can differentiate into common lymphoid progenitors (CLPs) and common myeloid progenitors (CMPs). CLPs and CMPs further differentiate into mature blood cells (4). LT-HSCs, ST-HSCs, and MPPs collaborate synergistically within the hematopoietic system, each playing a distinct yet complementary role in the production and maintenance of diverse blood cell lineages, collectively known as hematopoietic stem and progenitor cells (HSPCs).

Liggett and Sankaran have proposed models for HSC differentiation, including a “classical model,” a “continuum model,” and a “punctuated continuum model.” Particularly, the latter two models suggest different lineage biases in the differentiation of MPPs (5). In these models, MPPs are classified into four main groups: MPP1 are considered as ST-HSCs; MPP2 and MPP3 are myeloid-biased cells, which mainly differentiate into granulocytes and monocytes; and MPP4 refers to lymphoid-biased cells, responsible for the production of T, B lymphocytes, and NK cells (6). However, after 2 weeks of irradiation, HSCs temporarily overproduce myeloid-biased MPPs, accompanied by the fate of MPP4 reprogramming to the myeloid lineage, to support the expansion of myeloid cells and the rebuilding of the hematopoietic system (7). In addition, other pathological conditions such as inflammation (8, 9) and aging (10, 11) can also lead to a myeloid differentiation bias in HSCs. Beyond traditional flow cytometry, innovative single-cell multimodal approaches are revolutionizing the analysis of HSCs (12). Moreover, the single-cell transcriptome landscape of HSPCs is progressively being elucidated, offering crucial insights into the in-depth study of hematopoietic cell heterogeneity and developmental processes (13).

HSCs are considered to be the “roots” of the hematopoietic and immune systems, ensuring the proper functioning of critical physiological processes such as oxygen transport, hemostasis, immune defense, and tissue repair. The precise regulation of these processes is essential for maintaining health and is of paramount importance in disease treatment (14). However, the self-renewal capacity and differentiation potential of HSCs are not permanently maintained. With aging and interference from harmful external stimuli, HSCs undergo a process of senescence (15). For instance, chronic inflammation may cause a progressive and irreversible depletion of HSC function, resulting in a sustained inhibitory effect over time (16). Many features of HSC aging have now been identified, including a significant decrease in the number of quiescent HSC cells (i.e., G0 phase) (17); accumulation of DNA damage and mutations in HSCs (18–20); a decrease in self-renewal capacity; impaired hematopoietic reconstitution; clonal proliferation; and myeloid-biased hematopoiesis (21). Given the critical role of HSCs in hematopoiesis and immunity, investigating the factors that regulate their activity is of particular importance.

In recent years, the impact of diets on the body has been one of the research hotspots, and numerous studies have reported that different diets profoundly affect the functional homeostasis of HSCs. Specifically, in mice, a high-fat diet (HFD) refers to a dietary pattern in which fat provides at least 35% of the total energy intake (22), and can even reach up to 60% (23). This kind of diet often leads to obesity, indicating that mice are prone to excessive energy intake when fed an HFD. In contrast, in a normal diet, fat typically accounts for 10% of the energy intake (24). A 10-30% reduction in energy intake is defined as a moderate energy-restricted diet or dietary restriction (DR) (25). Severe energy-restricted diet (SERD) is commonly defined as a substantial reduction in daily caloric intake, such that it falls significantly below the threshold necessary to sustain basal metabolic functions and routine physical activities, and specifically, the energy restriction can exceed more than 40% of the normal caloric requirement (26–28). Here, we aim to provide an overview of the current understanding regarding the impact of HFD and energy-restricted diet on HSCs (**Table 1**). We anticipate that this review will not only clarify the existing knowledge but also inspire novel clinical applications, such as more refined nutritional interventions for patients with hematopoietic disorders, and ultimately lead to improved therapeutic outcomes and patient well-being in the clinical setting.

**Table 1:** Effects of HFD^*^ and Energy-Restricted Diet on HSCs^*^: phenotypes and mechanisms.

Dietary Intervention		Duration	Positive Effects on HSCs	Negative Effects on HSCs	Mechanism	Other Observed Changes	Model	References
HFD		10 days	Not detected	Inhibition of HSC production: Reduced HSC numbers due to increased bone marrow adipocytes.	PPARγ^*^ activation, adipogenic differentiation of BMSCs^*^	Increased bone marrow adipocytes, reduced osteoblasts; Increased DPP4^*^ secretion and the inhibition of bone regeneration capacity.	Mice (C57BL/6)	Ambrosi et al. (2017) (60)
		4 weeks	Not detected	Loss of HSC quiescence: HSCs exit the quiescent state and enter the cell cycle, leading to exhaustion. Myeloid bias: Increased differentiation toward myeloid lineage at the expense of lymphoid lineage.	LR^*^ clustering; TGF^*^-β/Smad2/3 signaling suppression	No significant changes in body weight or blood glucose. Increased LDL^*^-cholesterol levels in plasma;	Mice (C57BL/6 & congenic B6.SJL)	Hermetet et al. (2019) (38)
		6 weeks	Not detected	Loss of HSC stemness: Reduced ability of HSCs to self-renew. Myeloid bias: Increased differentiation toward myeloid lineage.	Gut microbiota alteration	Increased Body weight and body mass index; Reduced bone volume and trabecular number	Mice (C57BL/6)	Luo et al. (2015) (68)
		16+ weeks	Not detected	HSC dysfunction: Impaired self-renewal and differentiation capacity. Myeloid bias: Increased production of myeloid cells, leading to immune imbalance.	Chronic inflammation; Oxidative stress	Significant weight gain; Accumulation of pro-inflammatory cells; Increase in adipocytes in the bone marrow; Insulin resistance and abnormal glucose metabolism.	Mice (C57BL/6)	Bowers et al. (2021) (44)
		16+ weeks	Not detected	HSC dysfunction: Excessive proliferation of HSCs, leading to exhaustion. Myeloid bias: Increased production of inflammatory myeloid cells.	Cholesterol accumulation in lipid rafts	Significant increase in body weight; Atherosclerosis; Elevated total cholesterol and reduced HDL-C^*^; Inflammatory myeloid cell production	Mice (C57BL/6)	Murphy et al. (2014) (49)
Energy-Restricted Diet	DR^*^	3 weeks, 6 months, 1 year	Increased HSC quiescence: HSCs remain in a dormant state, preserving the stem cell pool. Improved repopulation capacity: Enhanced ability of HSCs to repopulate the bone marrow after transplantation.	Impaired lymphoid differentiation: Reduced production of lymphoid lineage cells (e.g., B cells, T cells).	Reduction in serum IGF-1^*^ levels	Weight loss; Increased red blood cells and hemoglobin; Impaired immune function	Mice (C57BL/6)	Tang et al. (2016) (99)
		Daily 10-hour feeding window for 6 weeks (after 6 weeks of HFD)	Reduce myeloid differentiation of HSCs: Reduced the number of MPPs^*^, Pre-GMs^*^, and GMPs^*^	Not detected	Reduction in the expression of the Cebpa gene (a transcription factor essential for myeloid differentiation in BM cells)	Reduced liver mass, fasting blood glucose, insulin, and insulin resistance index HOMA-IR^*^;	Mice (C57BL/6J)	Kim et al. (2022) (97)
		9 months	Rejuvenation of aging HSCs: Improved function of aged HSCs. Improved regenerative capacity: Enhanced ability to regenerate blood cells.	Impaired lymphoid differentiation: reduced CD150^low^ HSCs (lymphoid-biased HSCs)	UPR^mt*^ restoration	Reduced DNA damage signaling; Improved mitochondrial function; Reduced ROS^*^	Mice (C57BL/6J)	Tao et al. (2020) (17)
	DR (fasting)	24 hours of fasting	Improved HSC regeneration: Enhanced ability of HSCs to recover from stress.	Not detected	Inhibited Socs3^*^-mediated AKT/FOXO^*^-dependent signaling; Autophagy induction	Increased fatty acid oxidation; Decreased glycolysis; Reduced chronic inflammation	Mice (C57BL/6)	Dellorusso et al. (2024) (112)
		24 hours of fasting	Not detected	Inflammatory response upon refeeding: Increased inflammation after refeeding, potentially harming HSC function.	CXCR4^*^ upregulation	Increased serum corticosterone; Decreased blood glucose; Elevated free fatty acids	Mice (C57BL/6J)	Janssen et al. (2023) (115)
		48–120 hours of prolonged fasting	Enhanced HSC regeneration: Improved ability of HSCs to regenerate after stress. Increase the number of HSCs: LT-HSCs^*^, ST-HSCs^*^ are significantly increased.	Not detected	IGF-1/PKA* signaling reduction	Reduce DNA damage caused by chemotherapeutic drugs; Restore the lymphoid/myeloid cell ratio imbalance caused by chemotherapy. Enhance the survival rate of mice after chemotherapy.	Mice (C57BL/6J)	Cheng et al. (2014) (111)
	SERD^*^	5 weeks of low protein (2%) diet	Not detected	Cell cycle arrest in progenitor cells: Progenitor cells are halted in the G0/G1 phase, reducing hematopoiesis.	Downregulation of the cell cycle protein	Significant weight loss; Decreases in serum protein, albumin, and glucose; Reductions in insulin and IGF-1; Increased serum corticosterone.	Mice (C57BL/6J)	Nakajima et al. (2014) (130)
		5 weeks of low protein (2%) diet	Not detected	Impaired HSC function: Reduced LT-HSCs and ST-HSCs; Impaired CFU-GM^*^/CFU-M^*^ formation	Impaired MAPK pathway activation; Reduced hematopoietic transcription factors	Significant weight loss (20%); Reduced serum protein/albumin; Bone marrow hypoplasia	Mice (C57BL/6)	Santos et al. (2023) (134)
		Not specified (Knockout of nutrient status-related genes in mice for simulation)	Not detected	Depletion of HSC pool: Rapid exhaustion of HSCs.	mTORC1 activation due to the absence of SZT2 and TSC1; ROS production	Reduced blood cell types; Premature death	Mice (C57BL/6J (Vav^Cre^ transgenic mice)	Yin et al. (2022) (133)

Table annotations: The abbreviations used in the table and their full names are as follows:

*HFD, high-fat diet; *HSCs, hematopoietic stem cells; *PPAR γ, peroxisome proliferator-activated receptor γ; *BMSCs, bone marrow mesenchymal stem cells; *DPP4, dipeptidyl peptidase-4; *LR, lipid raft; *TGF-β, transforming growth factor-β; *LDL, low-density lipoprotein; *HDL-C, high-density lipoprotein cholesterol; *DR, dietary restriction; *IGF-1, insulin-like growth factor 1; *MPPs, multipotent blood progenitors; *Pre-GMs, pre-granulocyte/macrophage progenitors; *GMPs, granulocyte/macrophage progenitors; *HOMA-IR, homeostatic model assessment of insulin resistance; *UPR^mt^, mitochondrial unfolded protein response; *ROS, reactive oxygen species; *Socs3, suppressor of cytokine signaling 3; *AKT/FOXO, protein kinase B/forkhead box O; *CXCR4, C-X-C motif chemokine receptor 4; *LT-HSCs, long-term HSCs; *ST-HSCs, short-term HSCs; *PKA, protein kinase A; *CFU-GM, colony-forming unit - granulocyte-macrophage; *CFU-M, colony-forming unit – macrophage; *SERD, severe energy-restricted diet; *mTORC1, mechanistic target of rapamycin complex 1; *SZT2, seizure threshold 2; *TSC1, tuberous sclerosis complex 1.

## 2 Effects of HFD on HSCs

**Figure 1:**
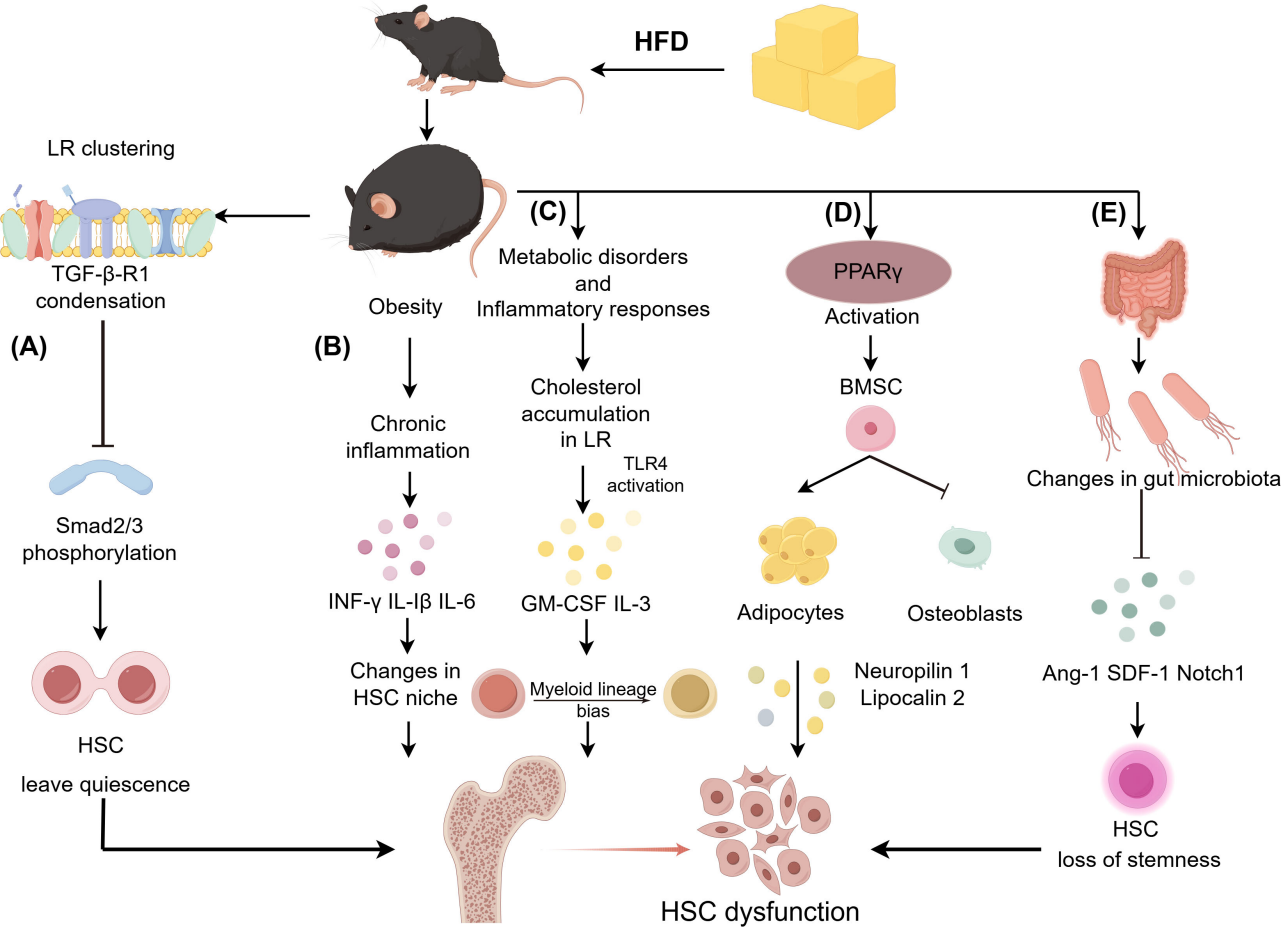
Mechanisms by which a high-fat diet (HFD) affects hematopoietic stem cells (HSCs). **(A)** HFD induces clustering of lipid rafts (LRs) on the cell membrane, thereby suppressing the transforming growth factor-β (TGF-β)/Smad2/3 signaling pathway, which in turn triggers the exit of HSCs from the quiescent state. **(B)** Obesity-related chronic inflammation caused by HFD changes the HSC niche. **(C)** HFD can cause the accumulation of cholesterol in LRs, thereby inducing the myeloid lineage bias of HSCs. **(D)** HFD promotes the adipogenic differentiation of bone marrow mesenchymal stem cells (BMSCs) by activating the peroxisome proliferator-activated receptor γ(PPARγ) signaling pathway, thereby inhibiting the production of HSCs. **(E)** HFD leads to alterations in the gut microbiota and the downregulation of related gene expressions detrimental to HSC homeostasis. (Created by Figdraw). *The full names of other abbreviations appearing in Figure: IFN-γ, interferon-γ; IL-1β, interleukin-1β; IL-3, interleukin-3; IL-6, interleukin-6; TLR4, toll-like receptor 4; GM-CSF, granulocyte-macrophage colony-stimulating factor; Ang-1, angiopoietin-1; SDF-1, stromal cell-derived factor-1.

Recent studies have reported that HFD significantly impacts the health of HSCs (**Figure 1**). Studies on the effects of HFD on HSCs are primarily conducted using mouse models. Both short-term and long-term HFDs trigger a transition in self-renewing HSCs towards differentiation into MPPs, and even enhance the ex vivo colony-forming ability of lymphoid and myeloid cells. Furthermore, prolonged exposure to HFD (18 weeks) significantly diminishes the hematopoietic reconstitution capability of HSC in mouse BM, indicating functional impairment of HSC (2). A 12-week HFD has been proven to have adverse effects on HSPCs within the interscapular brown adipose tissue (iBAT) and BM of mice, implicating a connection between HSPCs and metabolic dysfunction (29). In addition, long-term HFD increases the expression of hepatic insulin-like growth factor-1 (IGF-1) in mice, which may promote the activation of HSCs and exert deleterious effects (30). Berg et al. have reported that even after the removal of the obesogenic stimulus following long-term HFD, myeloid-biased progenitor cells continue to be produced, suggesting that the effects of long-term HFD on HSCs are difficult to reverse (2).

### 2.1 HFD induces an imbalance in HSC quiescence via lipid rafts

The quiescent state of HSCs plays a crucial role in maintaining their functional integrity and hematopoietic homeostasis. Appropriate quiescence serves to preserve the HSC pool by preventing exhaustion, regulates lineage differentiation balance, and ensures the stability of the hematopoietic system. Multiple mechanisms participate in the regulation of the HSC quiescence, including modulating cell cycle-related factors, transcription factor networks, cellular metabolic states, and signaling pathways related to the hematopoietic microenvironment (31). Among them, forkhead box O (FOXO) transcription factors and transforming growth factor-β (TGF-β) are two pivotal regulators of HSC quiescence and cell cycle control. Specifically, FOXO inhibits the expression of cell cycle proteins (such as Cyclin D) and promotes the expression of cell cycle inhibitors (such as p21 and p27), thereby preventing HSCs from prematurely entering the proliferative cycle. However, after the activation of the phosphatidylinositol 3-kinase - protein kinase B (PI3K-AKT) pathway, AKT induces a conformational change in FOXO protein through phosphorylation, thereby inhibiting its function in maintaining cell quiescence (32). Under physiological conditions, TGF-β acts as a guardian against HSC activation and cell cycle entry by engaging with TGF-β receptor type I (TGF-βR1), thereby maintaining the stem cell activity of HSCs. TGF-β regulates HSC cell cycle through multiple mechanisms, including upregulating cell cycle inhibitors such as p21 (33) and p57Kip2 (34), downregulating cytokine receptors such as interleukin-1 (IL-1) and granulocyte-macrophage colony-stimulating factor (GM-CSF), and interacting with the stromal cell-derived factor-1 (SDF-1) signaling pathway. These mechanisms collectively ensure that HSCs can maintain their quiescent state while also entering the cell cycle to proliferate in response to the body’s needs (35).

Additionally, lipid raft (LR), a membrane structure, plays a critical role in regulating HSC quiescence. LRs contain glycosphingolipids and protein receptors that form glycoprotein microdomains floating freely within the membrane bilayer (36). These LRs cluster signaling molecules with surface receptors such as C-X-C chemokine receptor 4 (CXCR4), the α4β1 integrin (VLA-4) that binds vascular cell adhesion molecule-1 (VCAM-1), and the c-kit receptor for stem cell factor (SCF). This molecular clustering is essential for retaining HSCs in the BM niche and maintaining their quiescent state. Evidence shows that inhibiting LR clustering leads to sustained elevation of FOXO nuclear concentration and induces murine HSC quiescence ex vivo (37).

Recent evidence has revealed that HFD modulates HSC quiescence through LR-mediated mechanisms. Research by François Hermet et al. has revealed that a short-term HFD (1˜4 weeks) leads to the formation of LR clusters on the membrane of HSCs and a pronounced condensation of TGF-βR1 within these LR clusters. This finding results in a consequent decrease in the phosphorylation of SMAD2/3, a downstream molecule of the TGF-β signaling pathway, indicating that HFD-induced condensation of TGF-βR1 in the HSC LR cluster leads to inefficient and impaired TGF-β-mediated signal transduction. This transduction, in turn, causes HSCs’ re-entry into the cell cycle and the loss of their ability to maintain quiescence (**Figure 1A**) (38). This effect in turn results in the altered transcriptional regulation of the aforementioned genes, thereby disrupting HSC quiescence (39–41).

Additionally, fms-like tyrosine kinase 3 (FLT3) plays a critical role in HSCs by regulating their proliferation and differentiation under physiological conditions, which is essential for maintaining hematopoietic homeostasis (42). Another study has shown that a short-term HFD induces the clustering of FLT3 receptors within LR on the membrane of HSCs, thereby enhancing the downstream JAK3/STAT3 signaling pathway and subsequently leading to a decrease in the content of primitive HSCs and an increase in the incidence rate of acute myeloid leukemia (AML) in a mixed lineage leukemia (MLL-AF9) knock-in mouse model (43). However, how short-term HFD affects LR content, and whether short-term HFD affects HSC function through other factors, needs to be further investigated.

### 2.2 HFD-induced chronic inflammation and metabolic disorders disrupt HSCs’ homeostasis

Long-term HFD (more than 16 weeks) leads to metabolic abnormalities such as obesity and hyperlipidemia. Obesity manifests as a chronic low-grade inflammation characterized by increased circulating levels of inflammatory factors such as tumor necrosis factor-α (TNF-α), interferon-γ (IFN-γ), interleukin-1β (IL-1β), and interleukin-6 (IL-6). The specific mechanisms by which inflammatory factor-related signaling pathways affect the HSC niche have been well elucidated (**Figure 1B**) (44, 45). Researchers have observed that activation of inflammatory pathways in HSCs leads to enhanced myeloid hematopoiesis with HSC expansion. Moreover, chronic inflammatory cytokine signaling can lead to HSC failure and may contribute to the development of hematopoietic malignancies, indicating that signaling pathways and BM niche impact both normal and malignant HSCs (46).

Inflammation activates associated signaling pathways, leading to oxidative stress in HSCs. The accumulation of ROS may disrupt the RAS-MAPK pathway- a signaling axis critical for maintaining the self-renewal capacity of HSCs through tightly controlled activation. This dysregulation subsequently induces excessive DNA damage and senescence, as well as apoptosis of HSCs (47). Spred1, a negative regulator of the RAS-MAPK signaling pathway, negatively modulates the self-renewal and adaptive capacity of HSCs under homeostatic conditions. However, under HFD conditions, Spred1 protects the homeostasis of HSCs, preventing HSC dysfunction induced by diet-induced systemic stress. Specifically, the protective effect of Spred1 on HSCs under HFD conditions is achieved by inhibiting the activity of the extracellular regulated protein kinases (ERK) and RhoA/Rho kinase (ROCK) pathway. ROCK pathway controls actin polymerization, which can lead to abnormal activation and proliferation of HSCs in Spred1-deficient mouse models (48).

When being exposed to HFD, the toll-like receptor 4 (TLR4) of HSPCs can be activated by sensing endogenous ligands such as damaging associated molecular patterns (DAMPs) (49), which are released during inflammation caused by hypercholesterolemia. This activation of TLR4 consequently inhibits the expression of apolipoprotein E (apoE) and ATP-binding cassette (ABC) transporters A1 and G1, which are indispensable for the efflux of cholesterol in cells. The insufficiency of these proteins results in the accumulation of cholesterol within the LRs of HSCs, and thereby promotes the activation of GM-CSF and interleukin-3 (IL-3) signaling, which aids in the proliferation of HSCs, especially in the production of inflammatory myeloid cells (**Figure 1C**). The production of inflammatory myeloid cells may drive their differentiation into macrophages with a pro-inflammatory phenotype, thereby exacerbating the development of atherosclerosis and forming a vicious cycle (50, 51). Furthermore, under hypercholesterolemia, sterol regulatory element binding protein 2 (SREBP2) activation is enhanced, and Notch1 is upregulated in human HSPCs, which contribute to HSPC emergence (52). However, exendin-4 (EX-4) prevents HSC proliferation induced by hypercholesterolemia via regulating cholesterol metabolism and inhibiting inflammation (53). In summary, we propose that cholesterol metabolism exerts a significant influence on the regulation of HSCs, offering new perspectives for the treatment of related diseases.

### 2.3 HFD modulates HSCs via BM niche remodeling

The BM niche is composed of a variety of cell types and extracellular matrix, including stromal cells, immune cells, and extracellular matrix. Specifically, it refers to the local tissue microenvironment in the BM that provides support and regulation for HSCs (54). A critical aspect of the BM niche is the dynamic balance between osteoblasts and adipocytes, which collectively govern HSC functional regulation. Bone marrow adipocytes (BMAs) secrete factors, such as neuropilin1 (55) and lipocalin 2 (56, 57), have been shown to impair hematopoiesis (58). Meanwhile, BM osteoblasts, considered as key regulators of the HSC niche, express SDF-1, Notch, and jagged 1 (Jag1) (59), facilitating HSC mobilization and differentiation and thus protecting hematopoiesis (23, 58, 60).

HFD leads to the activation of peroxisome proliferator-activated receptor γ (PPARγ), which increases the adipogenic differentiation of bone marrow mesenchymal stem cells (BMSCs). This results in overproduction of BMAs, which in turn inhibits osteoblastogenesis and negatively affects hematopoiesis (60) (**Figure 1D**). Consistently, long-term HFD increases adipogenic progenitor cells’ expansion in the BM, inhibiting HSC repopulation (61).

### 2.4 HFD influences HSCs by manipulating the gut microbiota

Gut microbiota has a tremendous influence on HSCs and immune homeostasis (62, 63). The metabolic byproducts of the gut microbiota, inflammatory signals, and their interactions with the BM niche have profound implications for the function and fate of HSCs (64, 65). HFD, as an energetic stressor, can indirectly affect gut microbiota, leading to a loss of HSC stemness (**Figure 1E**). In a healthy body, gut microorganisms promote hematopoiesis through complex mechanisms. In both mice and humans, distal gut microbiota is comprised of two major bacterial phyla: Bacteroidetes and Firmicutes (66, 67). A report showed that the abundance of Bacteroidetes and Firmicutes decreased in the cecum and ileum of mice after HFD, while the abundance of Verrucomicrobia, Proteobacteria, and Actinobacteria increased. Correspondingly, the expression levels of angiopoietin-1 (Ang-1), SDF-1, and Notch1 decreased, and the expression of kit ligand (KitL) increased, which was detrimental to HSC homeostasis. Moreover, the gut microbiota promotes myeloid-biased differentiation by enhancing the number and differentiation potential of GMP in the BM. Transplantation of gut microbes from normal mice was able to restore the adverse effects of HFD on HSCs (68). Moreover, the defective phenotype of aging HSCs is ameliorated by the fecal microbiota transplantation from young mice. This phenomenon indicates the significant importance of intestinal microbial barrier integrity for HSC function (69).

### 2.5 Impact of HFD on embryonic hematopoiesis

**Figure 2:**
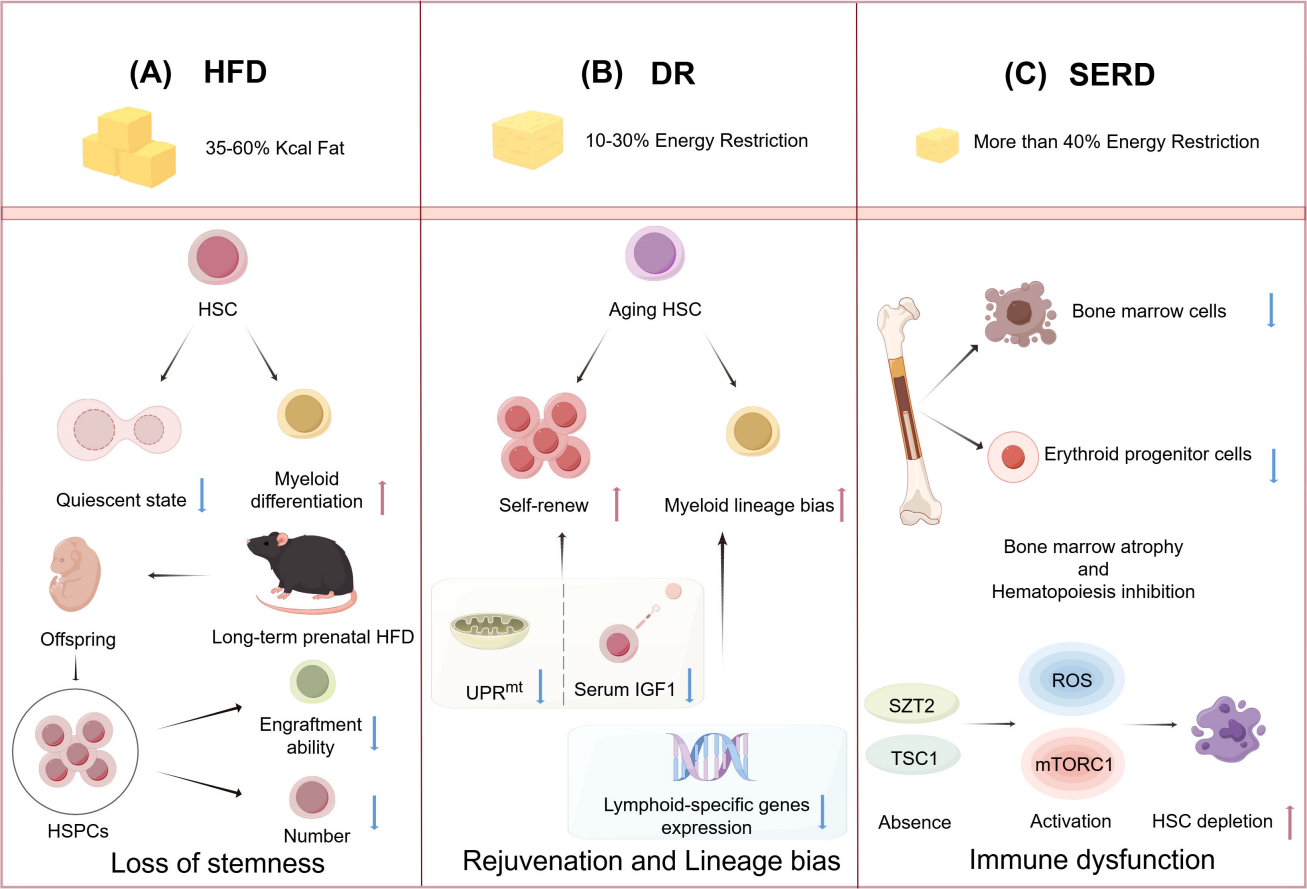
High-fat diet (HFD) and energy-restricted diet modulate the function of hematopoietic stem cells (HSCs). **(A)**: HFD causes HSCs to leave their quiescent state and generate myeloid differentiation. HFD feeding during pregnancy leads to impaired engraftment capacity and decreased numbers of HSPCs in offspring. **(B)**: Dietary restriction (DR) promotes the self-renewal of aging HSCs by reducing serum insulin-like growth factor-1 (IGF-1) levels and inhibiting the mitochondrial unfolded protein response (UPR^mt^). DR downregulates lymphoid-specific gene expression, which can lead to an increased myeloid differentiation of HSCs. **(C)**: Severe energy-restricted diet (SERD) leads to a reduction in the number of bone marrow (BM) cells and erythroid progenitor cells, resulting in BM atrophy and suppression of hematopoiesis. The absence of the seizure threshold 2 (SZT2) and tuberous sclerosis complex 1 (TSC1) leads to the activation of reactive oxygen species (ROS) and mechanistic targeting of rapamycin complex 1 (mTORC1) signaling pathways, which causes depletion of HSCs. (Created by Figdraw).

Epidemiologic and experimental evidence suggests that overweight mothers and long-term prenatal HFD can lead to fetal reproductive defects, growth restriction, hypothalamic developmental defects (70), cardiac abnormalities, and endocrine dysfunction (71–74). Studies have shown that in addition to its negative effects on the maternal BM HSCs and hematopoiesis, HFD also impacts the hematopoiesis of unborn offspring. A study of pregnant mice fed on HFD has demonstrated that a short-term HFD leads to an increase in HSPCs in fetal liver, but these fetal liver HSCs exhibit decreased proliferative potential. Meanwhile, long-term HFD in pregnant mice decreases fetal liver HSCs, and maternal weight loss cannot reverse it. Both pregnant and neonatal mice receiving HFD show a myeloid bias in HSCs, thus leading to hematopoietic dysplasia (75). It is worth noting that in another study, the impact of maternal obesity on offspring HSPCs was found to be sex-related. Specifically, male offspring exhibited reduced HSPC numbers and impaired engraftment, whereas female offspring showed no significant alterations in HSPC numbers or engraftment efficiency, but an increase in immune-related gene expression in HSPCs (76) (**Figure 2A**). Overall, these findings suggest that habitual intake of high-fat foods during pregnancy or prenatal obesity may impair the function of fetal liver HSCs. Late in gestation, the fetus establishes its initial immune function through hematopoiesis in the BM. However, this study did not focus on whether HFD negatively affects HSC function in the fetal BM of fetal mice, which requires further investigation.

### 2.6 Exercise reverses the detrimental effects of HFD on HSCs

There is evidence that exercise can reverse the negative effects of HFD on HSCs, primarily by altering the BM microenvironment, thereby benefiting hematopoiesis. Experiments conducted in both mice and humans have shown that exercise can increase the number of HSPCs in the BM and peripheral blood (77), and decrease BM adipose tissue (78). These phenomena have been associated with the regulation of adipogenic and osteogenic differentiation of MSCs. Mechanistically, exercise decreases the levels of miR-193 in extracellular vesicles within the BM of mice fed with HFD. These microRNAs are implicated in the inhibition of osteoblastogenesis (79). Specifically, high mobility group box protein-1 (HMGB1) is targeted by miR-193a to inhibit osteogenic differentiation of human BM-derived stromal cells (80), and the upregulation of miR-193b in HSCs can prevent the exhaustion of human HSCs by limiting proliferation and self-renewal (81). Moreover, a study also suggests that exercise reduces the production of inflammatory cells, as well as the abnormal proliferation and differentiation of HSCs (82). Adipose tissue is an important source of leptin in the BM microenvironment (83), and leptin promotes the maintenance of inflammation (84, 85). Exercise reduces leptin production in adipose tissue, thereby enhancing leptin receptor-positive BMSCs to produce hematopoietic factors that promote HSC quiescence. The reduction of leptin also increases CXCL12 expression (54, 86), which interacts with CXCR4 to anchor HSCs in the BM niche, thus facilitating the hematopoietic function of HSCs (87, 88). Additionally, researchers found that although exercise reduces circulating leukocytes in the blood, it does not lead to a decrease in the body’s resistance to external infections (78). Therefore, exercise not only reduces HFD-induced obesity and systemic inflammation but also mitigates the negative effects of HFD on HSC function.

## 3 Effects of moderate energy-restricted diet on HSCs

DR, also referred to as calorie restriction (CR), is a well-recognized anti-aging intervention that extends lifespan. Currently, there are differences in the specific experimental approaches to moderate energy-restricted diet across different studies. Omodei and Fontana have stated that experimental DR does not lead to nutritional deficiencies (25). In animal studies, control animals are typically fed an ad libitum (AL) diet. This controlled approach allows researchers to better understand the effects of dietary intake on health and longevity.

Current DR studies rarely specify precise limitations for the three major macronutrients (carbohydrates, proteins, fats). Research on the effects of a diet solely restricting carbohydrates on HSC function remains limited. Protein restriction may lead to protein-energy malnutrition (PEM) in mice and is excluded from typical DR research. Regarding fat restriction, limited studies show that a fat-free diet promotes HSPC mobilization (89, 90). However, the impact of fat restriction on HSC remains largely unknown. Based on existing evidence from DR studies, we propose that an effective moderate energy-restricted diet requires balanced, moderate limitation of all three macronutrients, aligned with normal dietary nutrient composition.

### 3.1 DR promotes HSC quiescence and rejuvenation

Abundant evidence demonstrates that DR is instrumental in delaying or even reversing immune aging and bolstering immune function (91). For instance, thymic immature T-cell precursors were more effectively preserved in mice subjected to DR compared to those with AL feeding, thus sustaining a stable reservoir of naïve T cells (92). Another study demonstrated that DR mitigated the accumulation of dysfunctional, aging T cells over time (93), implying a link between immune aging delay and DR. In addition, DR has been shown to enhance the protective function of memory T cells against secondary infections (94). Furthermore, a two-year DR regimen, reducing caloric intake by approximately 14% in a healthy cohort, was found to enhance human thymic function (95). DR has also been proven to alleviate the reduction in the diversity of the B cell receptor repertoire associated with aging in mice (96). Additionally, time-restricted feeding (TRF), characterized by a daily 10-hour feeding window followed by a 14-hour fast, has exhibited potential in preserving immune cell homeostasis in the BM and peripheral circulation, particularly in the context of obesity induced by HFD (97). Although the overall impact of DR on the immune system is perceived as beneficial, the precise mechanisms remain largely unelucidated.

Several studies have reported that DR can promote the repopulation of aging HSCs and enhance their functionality (**Figure 2B**) (98). DR has been shown to increase HSC quiescence and reduce their proliferation in response to stress, in contrast to the AL group (99). Furthermore, the duration of DR influences its effects on the function of HSCs. Short-term DR, lasting for 4 months, rejuvenated aging HSCs but did not significantly impact cell chimerism after BM transplantation. In contrast, long-term DR, extending for 9 months, not only rejuvenated aging HSCs and their associated hematopoietic progenitor cells but also enhanced cell chimerism following BM transplantation, indicating a substantial improvement in the body’s medullary hematopoietic function (17). This indicates that the long-term reconstitution capacity of HSCs is significantly influenced by the duration of DR. Still, there is a need for more systematic and comprehensive research to elucidate the impact of DR on HSCs.

The molecular mechanisms of how DR promotes the rejuvenation of aging HSCs are gradually being elucidated. DR has been observed to partially reverse the age-related increase in ST-HSCs, yet the impact of DR on HSCs varies among individuals with different genetic backgrounds (100). While DR ameliorates hematopoietic aging, this effect does not appear to rely on telomerase regulation (101). Improvements in HSC quiescence and regeneration under DR conditions are implicated to be linked to a decrease in serum IGF-1 level, as evidenced by the observation that IGF-1 supplementation reduces both the HSC proportion of G0 phase and the long-term reconstitution capacity to levels those observed with an AL diet (99). However, the precise molecular mechanisms through which IGF-1 regulates these HSC properties remain to be fully elucidated.

Mitochondria, as pivotal players in energy metabolism, significantly influence HSC functions. The mitochondrial unfolded protein response (UPR^mt^) has been shown to have negative effects on HSC homeostasis, which is activated during the transition of HSCs from quiescence to proliferation (102). Long-term DR has been proven to restore the expression of UPR^mt^-related genes, such as heat shock protein-10 (HSP10), HSP60, and sirtuin7 (SIRT7), to normal levels, thus promoting the rejuvenation of HSCs (**Figure 2B**) (17). Specifically, heat shock factor 1 (HSF1) is essential for the activation of mitochondrial chaperone protein genes, including HSP60 and HSP10. The occupancy of HSF1 is significantly enhanced during UPR^mt^ activation (103). SIRT7 has been identified as a key regulator of a branch of the UPR^mt^ pathway, with its inactivation leading to impaired HSC regeneration (104, 105). Furthermore, the accelerated accumulation of oxidized by-products with ROS stimulates mitochondrial dynamics through mitochondrial proteases and UPR^mt^ (106), which aggravates the aging of HSCs. Nevertheless, the precise mechanisms by which DR modulates HSC homeostasis via the alteration of mitochondrial function still require further investigation.

### 3.2 Fasting promotes the self-renewal capacity of HSCs

Fasting, as a form of DR, is one of the most prominent energy-restricted diet manipulation methods. This type of caloric restriction is typically achieved through periodic fasting, intermittent fasting, fasting mimicking diet (FMD, which includes very low calorie and low protein intake (107, 108). Current evidence indicates that fasting exerts pleiotropic effects on HSCs, enhancing their functional competence via coordinated modulation of self-renewal, stress adaptation, and lineage differentiation capacities.

Fasting has been recognized as a modality capable of promoting hematopoietic regeneration in both mice and humans (109, 110), which modulates metabolic homeostasis and mitigates inflammatory injury, thereby enhancing the functional capacity of HSCs. For instance, a study in mice indicated that prolonged fasting (lasting 48–120 hours) reduces circulating IGF-1 levels and protein kinase A (PKA) activity, thereby triggering signaling changes in LT-HSCs, which in turn modulate cellular stress resistance, self-renewal capacity, and balanced lineage regeneration (111). In another study, researchers demonstrated that aging-induced remodeling of the BM niche leads to suppressor of cytokine signaling 3 (Socs3)-mediated inhibition of the AKT/FOXO signaling axis, thereby suppressing glycolytic activity in HSCs. During this process, autophagy is activated as a protective mechanism to enable HSCs to adapt to this metabolic change and maintain quiescence. A short-term fasting (lasting 24 hours)/refeeding paradigm not only normalizes glycolytic flux but also enhances the regenerative potential of aging HSCs through autophagy induction (112). Additionally, regulation of inflammatory signaling may also be one of the important factors through which fasting affects the function of HSCs. The aberrant activation of the NLR family pyrin domain-containing protein 3 (NLRP3) inflammasome induced by mitochondrial stress regulates the functional deterioration associated with HSC aging (113). Since fasting has been proven to activate SIRT3 in monocytes, reduce the production of ROS, and thereby suppress the activation of the NLRP3 inflammasome and mitigate inflammatory responses in humans (114), we postulate that fasting may modulate HSC function via analogous mechanisms. However, additional experimental validation is necessary to confirm this hypothesis.

While fasting has traditionally been regarded as beneficial for overall health, emerging evidence suggests that cycles of fasting and refeeding may adversely affect peripheral immune function. Mechanistically, this immune impairment appears to be mediated through activation of the NLRP3 inflammasome and NF-κB signaling pathways, along with the induction of endotoxemia (114). Moreover, fasting triggers corticosterone-dependent Ly-6C^hi^ monocyte homing to BM via CXCR4 upregulation in mice, causing post-refeeding inflammatory bursts (115). Although direct evidence linking fasting/refeeding cycles to HSC dysfunction remains limited, the observed effects on peripheral immune cells suggest that such dietary interventions may not uniformly enhance immune competence. These findings underscore the necessity for more comprehensive investigations to elucidate the precise impact of intermittent fasting on immune regulation.

### 3.3 DR disrupts balanced lineage differentiation in HSCs

While DR has been shown to promote HSC regeneration-demonstrating its beneficial effects-emerging evidence suggests it may also disrupt balanced lineage differentiation (**Figure 2B**). For instance, Tang et al. demonstrated that the long-term DR promotes HSC quiescence while reducing CLP frequency, impairs lymphopoiesis, but enhances myeloid differentiation. However, supplementation with IGF-1 fails to restore balanced lineage differentiation, suggesting that the decline in IGF-1 is not the primary driver of the lineage differentiation imbalance observed under DR condition. Moreover, long-term DR progressively downregulates lymphoid-specific genes (e.g., Blnk, Rag1, IL-7Rα, and Ly6d) in lymphoid-biased HSCs within the BM of aged mice, sequentially impairing lymphoid lineage differentiation in a stepwise manner - first affecting CLPs (IL-7Rα^+^Flt3^+^c-Kit^mid/low^Sca-1^mid/low^lineage^−^), followed by pro-B cells (B220^+^CD24^+^AA4.1^+^TER-119^−^Gr-1^−^CD11b^−^CD3^−^), and ultimately compromising mature B cell development. However, no alterations in other immune cells, such as T cells or NK cells, are observed, highlighting lineage-specific impacts (99). These findings challenge the notion that DR universally delays immune aging, instead revealing its time-dependent detrimental effects on B-lymphopoiesis that contrast sharply with its protective thymic effects, suggesting DR may exert lineage-specific regulation of lymphocyte development through distinct mechanisms governing T-cell versus B-cell homeostasis, though the current lack of data on DR’s effects on BM T-cell precursors represents a critical knowledge gap in understanding its differential immune modulation.

DR-induced imbalanced lineage differentiation in HSCs has been confirmed by other studies. For example, long-term DR has been shown to more markedly decrease lymphoid lineage-biased HSCs (CD150^lo^ HSCs and CD41^-^ HSCs) (17). Consistently, fasting leads to the accumulation of naïve B cells in the BM. This phenomenon was attributed to the migration of peripheral naïve B cells into the BM, rather than an enhanced differentiation of B cells in the BM, because CLP, B220^+^ B cell precursors, as well as immature B (B220^+^lgM^-^lgD^-^) cells in the BM decreased during fasting (116). Additionally, research demonstrated that DR alters gut microbiota composition, increases intestinal butyrate levels, downregulates glycolytic pathway genes in BM B cells, reduces glucose uptake, and ultimately decreases lymphocyte counts (117).

Although DR impairs B-lymphoid differentiation and negatively affects the adaptive immune system, particularly in aging contexts - these effects are thought to be reversible. Dietary interventions such as refeeding represent effective strategies: when mice initially subjected to DR were subsequently returned to an AL diet, DR-induced myeloid bias in HSCs was reversed, while changes in myeloid differentiation genes remained insignificant (99). One study demonstrated that prolonged fasting/refeeding increases lymphocyte counts and reverses myeloid bias in mice (111). Additionally, IL-6/IL-7 supplementation has been shown to restore the impaired B lymphopoiesis in mice under DR, suggesting a link between cytokines and the hematopoietic changes (99). Besides, broad-spectrum antibiotic treatment (e.g. ampicillin) has been shown to reduce bacterial richness and the abundance of Lactobacillus and Bacteroides in DR mice, significantly increasing the number of CLPs, pro-B cells, and B cells (especially immature B cells) in the BM, with increased peripheral B and T cell levels (117). Another study showed that during starvation, serum leptin (a nutritional status sensor regulating hypothalamic energy homeostasis) decreases, while hypothalamic neuropeptide Y (NPY) increases, elevating corticosterone. After 48 hours of fasting, B-cell development in the BM was impaired, but intracerebroventricular leptin injection normalizes corticosterone levels and rescues B-cell defects. Oral administration of the glucocorticoid receptor antagonist RU486 has the same effect as intracerebroventricular leptin injection (118). Taken together, these results reveal that dietary or pharmacologic interventions represent promising approaches to restore DR-induced imbalanced lineage differentiation. Mechanisms regulating lymphoid differentiation in HSC are being further clarified—for instance, expression of key transcription factors like FOS, IKZF1, and KLF9 enhances lymphoid differentiation potential in human induced pluripotent stem cell (hiPSC)-derived hematopoietic stem and progenitor cells (iHSPCs). These findings may facilitate developing new strategies to improve lymphoid differentiation (119).

## 4 Effects of SERD on HSCs

SERD, which excessively restricts the body’s energy intake, may impair the body’s immune function. Nutritional intake is known to modulate immune cell activity through the action of adipokines. For example, adiponectin and leptin, both adipokines predominantly secreted by white adipose tissue, play pivotal roles in modulating immune functions. Leptin tends to promote inflammation, while adiponectin exerts a potent anti-inflammatory effect (120, 121). Under conditions of SERD, a decline in leptin levels is observed, while adiponectin levels remain stable. This imbalance can lead to a reduction in the number and functionality of effector immune cells (122). The compromised immune response associated with severely insufficient energy intake may also be partially due to a decline in medullary hematopoiesis.

SERD can lead the body to break down proteins for energy supply (123). If the body remains in severe energy deficiency for a long time, it can result in PEM (124). Protein serves as a crucial structural component for functional biomolecules, and its deficiency can adversely impact hematopoietic function (125). It is noteworthy that while the adequacy of protein intake from vegetarian diets has been a subject of debate, classic vegetarian diets are capable of providing enough protein for adult individuals (126). However, it is also observed that human vegetarians may be at a higher risk of being underweight (127). Mice on a 4% low-protein diet exhibited a reduction in BM cells, a depletion of progenitor cells, and an increase in cells in the G0/G1 phase. These malnourished mice also showed a decrease in reticulocytes, leukopenia, and indicators of anemia compared to the control group fed with a 20% protein diet (128). In another study, malnourished mice displayed severe BM atrophy, diminished expression of proliferating cell nuclear antigen, and evidence of gelatinous degeneration, as well as a reduction in hematopoietic tissue and an abnormal deposition of extracellular matrix components. This indicated a compromised hematopoietic microenvironment, which was further evidenced by a reduced capacity of the hematopoietic stroma to support the growth of HSCs (CD34^+^) *in vitro* (129). During PEM, the downregulation of cell cycle protein D1 inhibited the cell cycle in hematopoietic progenitor cells; however, this does not imply that PEM increases the quiescence of HSCs, but rather reflects the detrimental effects of protein deficiency on hematopoiesis (130). Furthermore, PEM leads to a reduction in erythroid progenitor cells, thereby causing anemia (131). These findings highlight the detrimental impact of PEM on the structural integrity and functional capacity of the hematopoietic microenvironment (**Figure 2C**).

PEM exerts a particularly pronounced impact on HSCs at the metabolic level. The homeostasis of HSCs is regulated by nutrient sensing and the mechanistic target of rapamycin complex 1 (mTORC1) signaling pathway (132). Seizure threshold 2 (SZT2) and tuberous sclerosis complex 1 (TSC1), as inhibitory molecules of mTORC1, play a crucial role in the homeostasis of HSCs during nutrient deficiency. Specifically, SZT2 is an essential protein for the downregulation of mTORC1 during nutrient scarcity, and its absence can lead to a reduction in HSC reserves and impaired regenerative capacity. Furthermore, the dual loss of SZT2 and TSC1 results in rapid exhaustion of murine HSCs, a decrease in all blood cell types, and premature death. Mechanistic studies indicate that the single loss of SZT2 or TSC1 only leads to a mild increase in mTORC1 activity and ROS production in HSCs, whereas the simultaneous loss of both results in a significant synergistic effect, causing a substantial increase in mTORC1 activity and ROS production, which rapidly depletes the HSC pool (133) (**Figure 2C**). PEM also compromises the activation of the MAPK signaling pathway in HSCs, with a marked effect on ERK1/2 activation, by perturbing intracellular metabolic homeostasis, particularly through disruptions in amino acid availability and energy supply. This metabolic dysregulation results in the suppression of the key hematopoietic transcription factors, such as GATA1/2, PU.1, C/EBPα, NF-E2, and IKZF3, thereby impairing the proliferative and differentiation capacities of HSCs and ultimately contributing to the pathogenesis of BM dysplasia (134). Elucidating the mechanisms by which PEM affects HSCs contributes to a deeper understanding of the metabolic regulation and functional impairments of HSCs under nutritional stress and provides potential therapeutic targets for the prevention and treatment of hematopoietic disorders caused by SERD.

## 5 Conclusion and perspective

In summary, the influence of different diets on HSC function is substantial. Excessive dietary fat is recognized as a detrimental stressor for HSCs, and exposure to HFD may result in the loss of HSC stemness, consequently diminishing their proliferative capacity and differentiation potential. Although DR may disrupt the balance between myeloid-biased and lymphoid-biased cells, it has been shown to promote the rejuvenation of aging HSCs. The precise mechanisms underlying these effects warrant further investigation. SERD, which can lead to PEM, also negatively impacts HSCs and the immune function of the body.

However, numerous critical questions remain unresolved. For example, how do high-sugar or high-protein diets impact the function of HSCs? Whether restricting a single nutrient category (e.g., carbohydrates, proteins, or fats) selectively affects HSC activity also requires clarification. Additionally, it remains unclear whether altering dietary patterns can promote HSC homeostasis, thereby contributing to the maintenance of organismal homeostasis. With advancing research, new dietary patterns have been shown to confer benefits to human health, offering new non-pharmacological avenues for promoting hematopoietic system health and managing related disorders (135). The dietary approaches to stop hypertension (DASH) diet, characterized by a low glycemic index and high nutrient density, enhances satiety and reduces food intake, thus aiding in the control of human obesity (136). The ketogenic diet (KD), meanwhile, mitigates endoplasmic reticulum stress, improving HFD-induced skeletal muscle insulin resistance (137). Notably, however, direct research on the effects of these alternative dietary regimens on HSC function remains scarce.

Furthermore, hematopoietic stem cell transplantation (HSCT) is a pivotal treatment for hematological diseases. Though the regulation of energy metabolism can significantly impact HSC function, clinical research in this field remains relatively limited, and numerous challenges persist. For instance, ensuring that patients adhere to energy restriction criteria without falling into severe energy deficiency and minimizing the negative effects on immune function are critical issues. For example, studies could explore how to enhance the self-renewal and regenerative capacity of HSCs by modulating energy metabolism, thereby improving the success rate of HSCT and reducing post-transplant complications. A multitude of studies have demonstrated a strong correlation between nutritional status and the prognosis of patients undergoing HSCT (138–140). Therefore, further clinical research on the energy metabolism regulating HSC function is urgently needed. Besides, omics analysis could be employed to assess the energy metabolism status of patients with hematopoietic system diseases, enabling the development of personalized treatment plans to optimize HSC function, ameliorate symptoms, and enhance therapeutic outcomes. Optimizing dietary intervention strategies to enhance post-transplant BM hematopoiesis and improve patient outcomes may open new auxiliary therapeutic pathways for the treatment of blood disorders, holding significant translational medical value.
